# Applying the Andersen behavioral model to the medication therapy management program: an approach for improving medication safety in older adults

**DOI:** 10.3389/fpubh.2024.1499362

**Published:** 2024-11-21

**Authors:** Yu-Hua Fu, Melissa Castora-Binkley, Antoinette B. Coe, Margie E. Snyder, Catherine E. Cooke, Carlyn E. Vogel, Lisa Hines, Alan Lyles, Nicole Brandt

**Affiliations:** ^1^Department of Practice, Sciences and Health Outcomes Research, University of Maryland Baltimore School of Pharmacy, Baltimore, MD, United States; ^2^Peter Lamy Center on Drug Therapy and Aging, University of Maryland Baltimore, School of Pharmacy, Baltimore, MD, United States; ^3^Pharmacy Quality Alliance, Alexandria, VA, United States; ^4^College of Pharmacy, University of Michigan, Ann Arbor, MI, United States; ^5^College of Pharmacy, Purdue University, Indianapolis, IN, United States; ^6^College of Public Affairs, University of Baltimore, Baltimore, MD, United States; ^7^Henry A. Rosenberg Professor of Government, Business, and Nonprofit Partnerships, University of Baltimore, Baltimore, MD, United States

**Keywords:** medication safety, chronic disease management, Medicare Part D Medication Therapy Management, Andersen Behavioral Model, access to healthcare, geriatric pharmacotherapy

## Abstract

Medication therapy problems (MTPs) are common among older adults and are associated with considerable morbidity, mortality, and healthcare costs. The Medicare Part D Medication Therapy Management (MTM) program, which includes Comprehensive Medication Reviews (CMRs), Targeted Medication Reviews (TMRs), and guidance on safe medication disposal, is designed to optimize therapeutic outcomes and reduce adverse events by addressing MTPs. Although this program has demonstrated success in reducing MTPs, its utilization remains low, with ongoing concerns about service access disparities, patient satisfaction, and long-term health outcomes. This perspective paper applies the Andersen Behavioral Model (ABM) to the Medicare Part D MTM program to enhance understanding of factors influencing service utilization and impact among older adults. The ABM provides a structured framework to examine how macro-and micro-level factors shape health behaviors and outcomes. By applying ABM framework to MTM, this paper highlights essential research directions to generate rigorous evidence for program evaluation, inform policy adjustments, and make targeted recommendations for improving MTM within the U.S. healthcare system. Furthermore, this work has potential implications for global programs aimed at enhancing medication safety by addressing MTPs and optimizing medication use.

## 1 Introduction

Medication therapy problems (MTPs) are prevalent among older adults (≥65 years), including issues related to appropriateness (e.g., unwarranted polypharmacy), effectiveness (e.g., subtherapeutic regimens), safety (e.g., adverse drug events, drug–drug interactions, and supratherapeutic doses), access, and adherence (1, 2). Factors such as age-related physiological changes, increased frailty, multiple coexisting conditions, and gaps in care across settings and providers for patients having complex medical conditions, significantly elevate the risk of adverse events in this vulnerable population (3, 4). Preventable adverse drug events contribute to substantial morbidity and mortality, often resulting in avoidable emergency department visits, hospitalizations, diminished quality of life, and unnecessary healthcare expenditures (5–9). Consequently, prioritizing medication safety initiatives for older adults is imperative and historical work can inform future global efforts to address preventable medication related harm (10–12).

In the United States (US), the Medicare Part D medication therapy management (MTM) program represents a strategic approach aimed at optimizing therapeutic outcomes and reducing the risk of adverse events and associated costs by identifying and addressing MTPs (13). This program was established under the Medicare Prescription Drug, Improvement, and Modernization Act of 2003 (Public Law 108–173) to benefit eligible beneficiaries, determined by their number of medications, chronic diseases, and Part D drug expenditures (14). Medicare Parts A and B comprise a federal fee-for-service health insurance program for individuals aged 65 or older and younger adults with disabilities (15, 16), while Part D specifically covers outpatient prescription drugs and related services for Medicare beneficiaries (16).

The Medicare Part D MTM program, overseen by the Centers for Medicare & Medicaid Services (CMS), targets high-risk populations vulnerable to MTPs (13, 17). Part D plan sponsors are required to offer MTM services to eligible beneficiaries namely an annual Comprehensive Medication Review (CMR), quarterly Targeted Medication Reviews (TMRs), and guidance on safe prescription medications disposal (17, 18). MTM programs have been shown to significantly reduce MTPs while improving clinical, economic, and humanistic outcomes (19–24). Over nearly two decades, the Part D MTM program has undergone continuous revisions to enhance service delivery, improve quality, and address gaps in care (13, 25). While studies show some success in reducing MTPs, healthcare utilization, and costs compared to standard care (26–28), challenges persist regarding overall service utilization, disparities in access, patient satisfaction, and the long-term effects on health and well-being (29–32).

To better inform research and policy aimed at enhancing MTM service utilization and effectiveness, it is essential to gain a deeper understanding of factors associated with utilization of MTM services and the impact of these services (33). In this perspective paper, we utilize the Andersen Behavioral Model (ABM) to examine the Medicare Part D MTM program, focusing on opportunities to improve our understanding of service utilization and its implications on medication safety for older adults (34).

## 2 Andersen behavioral model (ABM) applied to Medicare Part D MTM program

The ABM provides a structured framework for understanding how various factors influence health behaviors and outcomes (33, 34). Within the ABM, these factors are categorized into two primary domains—environmental factors and population characteristics—which further divided into five sections: external environment, healthcare system, predisposing characteristics, enabling factors, and need factors (34, 35).

**Figure 1:**
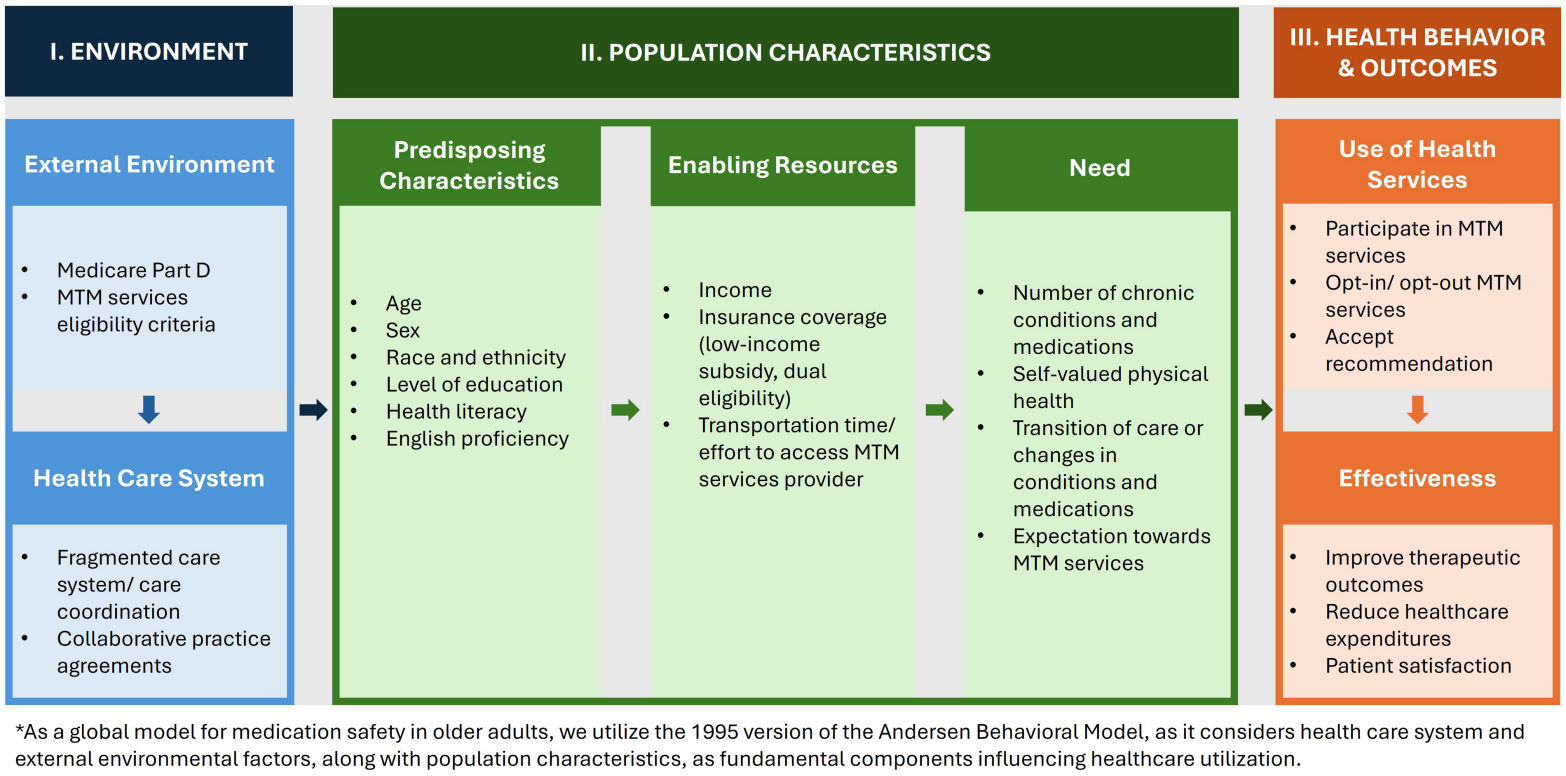
Illustration of Andersen behavioral model applied to medication therapy management (34).

By considering both environmental and individual-or patient-level (“population”) determinants of health behaviors, the ABM provides a valuable approach for assessing both macro-and micro-level influences on program participation (34). The following sections describe key elements of the ABM when applied to the Medicare Part D MTM program (see Figure 1). Additionally, Table 1 provides recommendations for a multifaceted approach to improve medication safety in older adults.

**Table 1:** Summary of research recommendations.

Domains	Recommendations
Environment	
External environment– Medicare Part D MTM policy	Research should focus on: How eligibility criteria changes affect the characteristics of eligible beneficiaries and their likelihood of receiving MTM services. Whether other criteria more appropriately identify beneficiaries, namely older adults, who could benefit from MTM.
Health care system	Research should focus on: Novel approaches to bridge the disconnect, and explore ways to overcome barriers at multiple levels, and foster collaboration between all key stakeholders Impact of CPAs on MTM utilization and effectiveness.
Population characteristics	
Predisposing characteristics (e.g., race, ethnicity, age, gender, marital status, education level)	Research should focus on: Strategies to mitigate the variable of MTM eligibility criteria on disparities. Understanding the barriers to receiving CMRs in different racial and ethnic subgroups and strategies necessary to address gaps.
Enabling resources: income, health insurance, region of residence	Support MTM providers collaborating with social work professionals and community health workers to tackle broader social determinants of health, such as food insecurity, job opportunities, and transportation barriers for physician visits Conduct ongoing evaluation of telehealth programs to assess equity, access and effectiveness to ensure value to the patient, caregiver and program.
Need: health status perceived by patients	Developing personalized approaches to assist during clinically significant moments (e.g., transitions of care) and highlighting the benefits of MTM services can increase awareness and perceived need, ultimately enhancing participation in these services.
Health behavior and outcomes	
Use of health services	Research should focus on: Understanding how environmental factors and population characteristics impact MTM service utilization and to what extent.
Effectiveness	Develop and implement new quality performance measures including patient-reported outcome performance measures to assess the impact and quality of MTM programs

CMR, comprehensive medication review; MTM, medication therapy management.

### 2.1 Environment

#### 2.1.1 External environment– Medicare Part D MTM policy

Although a later addition to the initial ABM, the external environment was recognized as having an important impact on health services utilization (34). Environmental factors include physical (health organizations), political (health policy), and economic (healthcare resources) components (33, 34). Particularly in the context of the Part D MTM, health policy changes have led to differential impacts on utilization of MTM services.

Specifically, CMS sets MTM program eligibility criteria using minimum and maximum thresholds per patient for number of medications and number of chronic diseases, along with a minimum annual Part D drug costs threshold to qualify for services (36). Moreover, Part D plan sponsors have historically been allowed to specify additional targeting criteria which has led to fewer eligible beneficiaries (36–38). This eligibility and targeting criteria have changed consistently throughout the history of the program, impacting who has the opportunity to receive these medication management services (36, 37).

In 2012, enrollment of beneficiaries into the MTM program by plan sponsor ranged from less than 0.2 percent to more than 57.0 percent, largely determined by higher versus lower eligibility threshold levels, respectively (39). For instance, when Part D plan sponsors only required the presence of two chronic conditions, enrollment was 16.4 percent (39). When this threshold was set at 3 chronic conditions, enrollment was lower at 9.2 percent (39). Similarly, when plan sponsors allowed the use of any Part D drug, enrollment was 12.7 percent compared to an enrollment of 4.4 percent when the use of drugs from specific medication classes were required (39).

With the option to refine targeting criteria, plan sponsors often set more stringent thresholds, likely driven by the incentives to limit access stemming from the absence of reimbursement for MTM services under Part D, as well as the disincentive to promote access linked to quantity-focused measures like the CMR completion rate assessed by CMS (39–42). This had led to only 8% of beneficiaries being eligible for MTM services in 2020 (40).

To partially address these limitations, CMS issued a final rule in April 2024, updating eligibility and limiting plan sponsors’ flexibility in allowable targeting criteria (38). This policy, effective from January 2025, is anticipated to nearly double MTM eligibility from 3.6 million to 7.1 million beneficiaries (43). Under the new guidance, beneficiaries must meet a minimum threshold of 2 to 3 of all 10 CMS-defined chronic diseases (Alzheimer’s disease, bone disease-arthritis, diabetes, heart failure, dyslipidemia, respiratory disease, hypertension, end-stage renal disease, mental health, and HIV/AIDS), fill a minimum of 2 to 8 covered Part D maintenance drugs, and anticipate incurring a designated annual drug cost based on the average for 8 generic covered Part D drugs, a substantial reduction in cost thresholds compared to previous years (40, 43, 44).

As health policy changes directly impact the MTM program, future research should focus on how changes such as these effect beneficiaries’ eligibility and likelihood of receiving MTM services. Additionally, research opportunities remain to determine optimal eligibility criteria to more appropriately identify beneficiaries, namely older adults, who could benefit from MTM services.

#### 2.1.2 Health care system

Factors related to the health care system itself should also be considered within the ABM framework to fully understand service utilization as it pertains to environmental influences (34). From how healthcare systems are organized (or disorganized) to the presence or absence of specific provider types and facilities, the healthcare environment contextualizes health services use (33, 34). The U.S. healthcare system, often characterized as fragmented, is typically operated in silos where physicians are not accustomed to providing coordinated and collaborative care (45–48).

Not unexpectedly, MTM providers and prescribers frequently experience limited care coordination, which hinders the uptake and diminishes potential benefits of the MTM programs (45, 46). A key challenge is the lack of direct communication and collaboration between prescribers and MTM practitioners, typically pharmacists, who perform the CMR (49–51). These practitioners often work for community pharmacies, MTM vendors, or Medicare Part D plan sponsors, rather than within the prescribers’ practice (49–52). This separation limits the integration and effectiveness of MTM services, as pharmacist’s recommendations may not be seamlessly communicated to or implemented by the prescriber (53, 54).

Opportunities to improve utilization of MTM services are through Collaborative Practice Agreements (CPAs), which allow pharmacists to adjust medication regimens and order relevant tests to monitor medication use and ensure safety. CPAs vary state by state in terms of scope and protocol but afford ways to reduce fragmentation and burden (55, 56). Without CPAs, the burden may fall on patients to communicate MTM recommendations to their prescribers, further complicating the care process (48, 55).

Limited care coordination and sharing of medical information between prescribers and MTM practitioners hampers the personalized nature of MTM care (53, 54). Without stronger collaboration and trust between patients, MTM practitioners, and prescribers, the full potential of MTM services is unlikely to be achieved (47, 48). Novel approaches are needed to bridge this communication gap, and further research is essential to explore effective strategies to overcome barriers at multiple levels and foster collaboration between all key stakeholders (49, 58).

### 2.2 Population characteristics

#### 2.2.1 Predisposing characteristics

According to the ABM, predisposing characteristics form the foundation for differences in an individual’s ability and propensity to utilize health care services (34, 35). Predisposing characteristics are more commonly thought of today as sociodemographic variables like age, education, race and ethnicity (59). Andersen described inequitable access to care as care that was determined based on such predisposing factors (34).

Studies have shown that, despite some improvements, disparities in access to and utilization of MTM services among racial and ethnic subgroups have persisted since the inception of the program (49). While the eligibility criteria, which has been based on number of medications, chronic diseases, Part D drug expenditure, identify at-risk beneficiaries many individuals at high risk for MTPs, they fail to account for differences in healthcare utilization across racial and ethnic subgroups with the same medical conditions (60–64). Studies have found that minorities, such as Black and Hispanic beneficiaries, are less likely to meet Medicare’s minimum thresholds for MTM eligibility compared to White beneficiaries (65, 66). Asian, Hispanic, and Native American beneficiaries are less likely to receive a CMR after being offered the service compared to White beneficiaries (65, 67, 68). Additionally, it typically takes longer for Black, Asian, and Hispanic beneficiaries to receive a CMR after enrolling in MTM services compared to White beneficiaries (65).

Addressing these disparities requires a multifaceted approach that may include refining eligibility criteria, understanding and overcoming barriers to receiving services, such as health literacy and language proficiency, specific to cultural preferences, and focusing on the needs of underserved communities (40, 69–71).

#### 2.2.2 Enabling resources

The ABM describes enabling resources as both community-level and individual-level factors that influence the likelihood of service utilization (34). Community-level factors refer to the presence of health professionals and facilities, which can vary significantly between communities (33). This variation directly impacts the likelihood of service utilization. Previous studies have noted that individuals residing in areas with limited primary care resources or healthcare professional shortages face significant challenges in accessing MTM services, reducing their likelihood of participation even when eligible (70, 72). As Part D MTM services have transitioned from face-to-face interactions to being predominantly delivered via telephone or other electronic means (51, 52), the traditional understanding of community-level resources may be evolving, particularly in today’s telehealth landscape (73, 74).

Individual-level factors include indicators of economic status, such as Medicare-Medicaid dual eligibility, food insecurity or low-income subsidy (LIS) status (59). These factors can either promote or prohibit individuals’ likelihood to receive health care services (34). Studies have reported associations between these economic indicators and both MTM eligibility and CMR completion (59, 68, 75). Beneficiaries with LIS or those who are Medicare-Medicaid dual-eligible often represent a vulnerable population, facing higher healthcare costs, greater medication utilization, and increased rates of high-risk medication use (76). They also demonstrate lower persistence and adherence compared to non-LIS or dual-eligible beneficiaries (76). Previous literature highlights challenges in engaging LIS and Medicare-Medicaid dual enrollees, including lower rates of MTM service uptake and higher rates of opting out of services (68, 75).

Racial and ethnic minorities, who are disproportionally affected by low-income status in the US, often face additional barriers accessing their healthcare needs, despite targeted support from CMS policy (68, 77, 78). To address these challenges, further efforts should explore how MTM providers can collaborate with social work professionals and community health workers to tackle broader social determinants of health, such as food insecurity, job opportunities, and transportation barriers for physician visits (78, 79). Additionally, the ABM can be used to further understand the impact of enabling factors on the utilization and effect of MTM services.

#### 2.2.3 Need

Perceived need plays an important role in the use of health care services (33). How individuals view their own health, their functional state, and how they experience symptoms and complications influence whether they view their condition as requiring assistance or guidance from healthcare professionals (33, 34). The ABM focuses this concept of need based on a patient-centric approach for help-seeking rather than based on a health professional’s determination of need (34). Additionally, the ABM also recognizes the influence of environmental and predisposing factors on the perception of need (34).

Part D MTM eligibility and targeting criteria have primarily addressed “objective” needs, such as numbers of chronic conditions and medication, and drug spending for at risk beneficiaries (40, 43). However, these criteria overlook “subjective” needs, such as patients’ perceptions of their health status (80). Previous literature has highlighted that patients’ perceptions of their health status significantly influence their interest in receiving MTM services (81). For example, patients with a higher number of prescribed medications or concerns about potential adverse effects are more likely to seek a CMR with a pharmacist (81). Beneficiaries have reported that MTM services are particularly valuable during clinically significant moments, such as when starting new medications, adjusting existing medications, or following recent health changes and/or transitions in care (77).

Low participation in Part D MTM services may be due to patients’ lack of awareness about their eligibility, a perception that they do not need the service, or low expectations of its value (82). Novel strategic patient outreach targeting MTM-eligible populations could significantly benefit those currently unaware or uncertain about MTM services (49). Studies have shown that patients are motivated to participate in MTM for reasons such as gaining better understanding of their medication therapy, reviewing the efficacy of their medications, potential cost savings, and benefiting from the pharmacist’s expertise (20, 83). They also value personalized information about their medications (83). Targeted education initiatives, such as presentations in patient-friendly language to local older adult community groups or marketing by community pharmacies that provide MTM services, could further boost awareness, understanding, and motivation for MTM engagement (58, 81).

### 2.3 Health services use and outcomes

#### 2.3.1 Use of health services

The original ABM focused solely on utilization as the main outcome but was later expanded to include important outcomes of care like patient satisfaction and effectiveness (34, 35). While the literature described here for Part D MTM reports factors individually related to their impact on MTM participation, this may be the greatest opportunity for further research as there is a lack of consensus regarding influences on who ultimately utilizes MTM services and the effectiveness of MTM services (84).

#### 2.3.2 Effectiveness

For example, systematic reviews have indicated that MTM reduced some MTPs, such as inappropriate use, nonadherence, and medication costs, but the heterogeneity of the studies reviewed was a concern limiting the ability to make confident conclusions about the impact of MTM services (26, 29, 85). Furthermore, a five-year demonstration (2017–2021) evaluated whether flexibility in MTM program design along with payment incentives for Part D sponsors could improve therapeutic outcomes and reduce Medicare expenditures (86). The model did not show a significant overall impact on net Medicare Parts A and B expenditures or intermediate measures of medication use (e.g., adherence) (77). It did, however, illustrate a different approach to MTM, with some services designed around a beneficiary’s unique needs such as during transitions in care, a time for heightened medication-related concerns (77).

## 3 Discussion

Applying the ABM allows for a critical assessment of the factors that affect MTM service utilization and related impact on outcomes (34). These insights have the potential to inform research and policies that aim at improving the MTM program, which can serve as a global model for enhancing medication safety among older adults. While the ABM highlights several key factors, it also recognizes the need for further consideration of additional elements (34). This review provides a foundation for selecting variables associated with MTM service use, with Figure 1 serving as a starting point for identifying the factors that influence this utilization according to the ABM.

The ABM emphasizes the importance of understanding what matters to the patient (34). Patient-reported outcome or experience measures could be used as outcomes in the ABM framework (34). Patient-reported outcomes are directly reported by patients using validated tools, allowing for self-assessment of health-related quality of life, functional status, symptom and symptom burden, health behaviors, or experience with care (25, 87). Patient experience measures focus on aspects of care such as communication with clinicians, responsiveness of staff, ease of scheduling an appointment, spending enough time with the patient, and explaining things in ways the patient can understand (87, 88).

The ABM was initially developed in the 1960s to enhance the utilization of healthcare services (34, 35). Although it was established during an era when healthcare costs were less of a concern, disease burden was significantly lower, and society’s relationship with health professionals were quite different compared to today, the ABM remains relevant in the context of the Part D MTM program (34). This program has been underutilized (52), yet as a preventive service, it has the potential to greatly benefit eligible beneficiaries and should be taken advantage of. However, as described previously, equitable access and uptake of this preventive service continue to be challenged.

Gaining a clearer understanding of individuals’ likelihood of participation in the Part D MTM program is one step toward connecting these influences on specific outcomes. It should be recognized that understanding the relationships between the factors and outcomes may lead to implications that have varying degrees of actionability (33). For example, environmental and predisposing factors are often very difficult to change whereas enabling factors and perception of need may be more modifiable.

## 4 Conclusion

This perspective paper applied the Andersen Behavioral Model for Health Services Use to the Medicare Part D MTM program, which provides a guide for needed research in generating rigorous evidence for program evaluation to inform additional policy changes and to make specific recommendations for interventions to improve the service. The application of this framework seeks to ensure that MTM programs meet the needs of older adults, thereby improving medication safety. In the long term, we hope this framework can be applied not only to the Medicare Part D MTM program within the U.S. healthcare system but also to global programs aimed at improving medication safety by addressing MTPs and optimizing medication use.
